# The Incidence of *Dirofilaria immitis* in Shelter Dogs and Mosquitoes in Austria

**DOI:** 10.3390/pathogens10050550

**Published:** 2021-05-02

**Authors:** Karin Sonnberger, Hans-Peter Fuehrer, Bernhard Werner Sonnberger, Michael Leschnik

**Affiliations:** 1 Clinical Unit of Internal Medicine Small Animals, Department/Universitätsklinik für Kleintiere und Pferde, University of Veterinary Medicine Vienna, Veterinärplatz 1, 1210 Wien, Austria; michael.leschnik@vetmeduni.ac.at; 2 Department of Pathobiology, Institute of Parasitology, University of Veterinary Medicine Vienna, Veterinärplatz. 1, 1210 Wien, Austria; Hans-Peter.Fuehrer@vetmeduni.ac.at (H.-P.F.); bw.sonnberger@gmail.com (B.W.S.)

**Keywords:** dirofilariosis, zoonosis, vector borne, mosquitoes, canine heartworm disease

## Abstract

To estimate the incidence of *Dirofilaria immitis* in Austrian shelter dogs and mosquitoes trapped in their proximity, 115 shelter dogs from fourteen animal shelters located in five different Austrian states were examined. Blood samples were screened for *D. immitis* using ELISA antigen-testing, PCR and microscopical examination for microfilariae. In total, 91% of the dogs originated from countries endemic for dirofilariosis. Eleven dogs (9.6%), all originating from Hungary, tested positive for *D. immitis*. None of the dogs examined showed microfilaremia. Eight dogs showed no or only mild clinical signs (e.g., infrequent coughing), and three dogs showed frequent coughing, dyspnea, exercise intolerance, blunt fur or weight loss. In total, 205 Mosquitoes of ten different species were caught at five different shelter sites in four different Austrian states, using CO_2_-baited mosquito traps set once a month (June–September 2019) for 24 h. All 205 mosquitoes tested negative for *Dirofilaria* spp. via PCR. The risk of endemisation of *D. immitis* in Austria (and other non-endemic countries in a similar situation) is very serious and its zoonotic potential should be communicated more strongly. To monitor a possible transmission of microfilariae from untreated or even untested positive dogs, e.g., in animal shelters, to mosquitoes in the near surroundings, frequent screening for *Dirofilaria* in mosquitoes should be used more intensively. Current knowledge on *D. immitis* should be integrated into daily veterinary practice and dog owners should be proactively educated, especially before traveling to endemic areas or adopting dogs from endemic countries. Animal shelters and animal welfare organizations should be provided with appropriate education and veterinary guidance regarding the testing and treatment of dogs imported from high-risk areas.

## 1. Introduction

*Dirofilaria immitis*, commonly known as “canine heartworm”, is a filarioid parasite transmitted by mosquitoes and the causative agent of a zoonotic disease, canine cardiopulmonary dirofilariosis [1]. The parasite can be introduced to non-endemic countries by dogs with a travel history to endemic regions, as well as dogs imported from endemic countries [2,3]. Noteworthy in this context is the increasing number of dogs matching these criteria [4,5].

There are several mosquito species in Austria that can act as vectors for *D. immitis* as well as *D. repens* (the causative agent of canine subcutaneous dirofilariosis). Additional competent species might show an increasing occurrence in Europe because of the proceeding climate change, potentially enabling autochthonous transmission in previously unaffected countries [1,3,6,7]. The occurrence of *D. repens* could be considered a model for the estimated future distribution of *D. immitis*, as the heartworm seems to be spreading mainly to the areas where *D. repens* has already established itself [4,8]. The first autochthonous findings of *D. repens* in mosquitoes in Austria were made in 2012, and seven cases of cutaneous dirofilariosis in dogs were thought to be autochthonous infections until 2014, while the first autochthonous *D. repens* infection in humans was found even earlier, in 2006 [9,10,11]. By 2014, a total of 25 infections with *D. immitis* had been reported in dogs in Austria. The annual number of reported cases then rose significantly until 2018 [5]. Since 1978, three infections with *D. immitis* have also been reported in humans in Austria, causing pulmonary symptoms in the patients [12].

The diagnosis of canine cardiopulmonary dirofilariosis should be based on a thorough clinical examination and a combination of different diagnostic tools, such as screening for microfilariae in the blood, thoracic radiography, cardiac ultrasound, ELISA antigen-testing or PCR [13,14]. Clinical examination and clinical staging are essential, especially to determine the appropriate treatment protocol. Three stages are distinguished in the clinical course of canine cardiopulmonary dirofilariosis. The first stage is an asymptomatic infection or expression of very mild symptoms, such as exercise-induced dyspnea, occasional coughing and mild apathy. Chronic coughing, dyspnea, exercise intolerance, blunt fur, weight loss, mild anemia and frequent vomiting usually characterize a second-stage infection. Following a longer course of disease with a large worm burden and symptoms such as tachycardia, tachypnea and syncope in addition to the symptoms seen in stage two, the infection can be classified as stage three [15,16].

Asymptomatic courses of canine cardiopulmonary dirofilariosis are very common. Many dogs show no symptoms, even after an incubation period of at least five to six months, so this can be a huge problem in the successful monitoring and prevention of the introduction of *D. immitis* from endemic to non-endemic countries [17]. An accurate and consistent screening program for dogs imported from endemic countries or dogs with a travel history to endemic regions is therefore essential to prevent the establishment of dirofilariosis in non-endemic countries.

The Federal Ministry of Social Affairs, Health, Long-Term Care and Consumer Protection (Bundesministerium für Soziales, Gesundheit, Pflege und Konsumentenschutz, BMSGPK, Vienna, Austria) listed 37 approved animal shelters in 2017 [18]. In 2002, 2153 dogs were accommodated in Austrian animal shelters, accounting for 11% of the shelter animals cared for. The average duration of residency of the dogs was six months. All animal shelters reported an occupancy rate of 100%. Considering that 70.8% of the shelters were also classified as financially poor and the average annual veterinary costs amounted close to EUR 107 per animal, it is comprehensible that routine screening for travel diseases was not part of the standard procedures [19].

This study aimed to estimate the incidence of *D. immitis* in shelter dogs (including a high percentage of imported dogs), as well as in competent vector mosquitoes in Austria, by screening dogs and mosquitoes in animal shelters in different Austrian federal states.

## 2. Results

### 2.1. Dogs

#### 2.1.1. General Findings

Included in this study were 115 dogs living in fourteen animal shelters in five Austrian districts. Fifty-nine dogs were female and 56 male. Nineteen dog breeds were represented; however, 64% of the dogs were cross-bred. The average age of the dogs was 5.3 years. At the time of the examination, the duration of the dog’s stay in the shelter ranged from one week to seven years. According to the shelter managements, all dogs included in this study were treated prophylactically with macrocyclic lactones (milbemycin oxime) at a minimum frequency of four times a year.

#### 2.1.2. Country of Origin

The 115 dogs originated from eleven different countries or were of unknown origin (18.3%). In total, 91% of the dogs originated from countries endemic for *D. immitis*. An overview of the countries is given in Table 1.

#### 2.1.3. Blood Smears

No dogs showed microfilariae in the blood smears.

#### 2.1.4. ELISA Antigen-Testing

Of the 111 dogs screened with an ELISA rapid test kit, six dogs tested positive for the *D. immitis* antigen, two dogs for *Borrelia burgdorferi* antibodies and eight dogs for *Anaplasma phagocytophilum/platys* antibodies. The dogs that tested negative were re-tested using heated serum samples to exclude false-negative results. Of the dogs tested via an external laboratory, five were positive for the *D. immitis* antigen. The results of the blood tests of dogs positive in at least one assay can be found in Table 2.

#### 2.1.5. PCR

Only one of the samples previously tested positive in ELISA antigen-testing also tested positive in the PCR assay. No false-negative ELISA antigen-tests were found by PCR. The results of the blood tests of dogs positive in at least one assay can be found in Table 2.

#### 2.1.6. Clinical Staging

The clinical staging of the *D. immitis* infected dogs (*n* = 11) resulted in eight dogs without clinical signs or with mild signs (stage one), three dogs showing clinical signs consistent with stage two and no dogs in stage three. Twelve dogs were examined by echocardiography and macrofilariae could be found in the right ventricle or pulmonary arteries in three dogs. Thoracic radiography was performed in twelve dogs, showing no abnormalities.

### 2.2. Mosquitoes

#### 2.2.1. General Findings

In total, 205 mosquitoes were caught in all five locations in June–September 2019. Most mosquitoes were found during July at the location No. 3 (Table 3).

Most mosquitos found were specified as *Culex pipiens/torrentium* (*n* = 185). Twenty individuals from nine other species were identified (Table 4). Seven of all ten species found had been proven or thought to transmit *Dirofilaria* spp. (*Ae. vexans, An. maculipennis* complex, *An. plumbeus*, *Cq. richardii*, *Cx. pipiens/torrentium*, *Cs. longiareolata*, *Oc. caspius*), and accounted for 202 individuals (98.5%).

#### 2.2.2. PCR

All 205 mosquitoes tested negative for *Dirofilaria* spp. via PCR.

## 3. Discussion

Infections with the zoonotic parasite *Dirofilaria immitis* are of growing concern in Central Europe, as *D. immitis* has shown a rapidly increasing prevalence over the past few years. Unfortunately, the attention from the public at large and the veterinary profession, in particular, did not increase to the same extent [20,21,22].

Originally native to tropical or subtropical climates, *D. immitis* can be seen emerging in adjacent countries with rather mild climates. In Europe, the parasite is predominantly found in southern countries, such as France, Greece, Italy, Portugal and Spain. A particularly strong increase in the number of infections is currently observed in Eastern Europe [4,23]. As climate change and globalization proceed, additional competent invasive mosquito vectors, such as the Asian tiger mosquito (*Aedes albopictus*), may become established in previously unaffected countries, facilitating autochthonous transmission of *D. immitis* [1,3,6,7,24,25]. Dogs with a travel history to endemic regions, as well as dogs imported from endemic countries, can introduce the parasite to non-endemic countries. Therefore, these dogs are of particular epidemiological interest [2,3]. Sixty-seven of the 115 dogs included in this study originated from abroad, 91% of these from countries known to be endemic for *Dirofilaria* spp. Twenty-seven dogs were born in Austria and never left the country, while 21 dogs were of unknown origin. Besides Austria, most of the dogs came from (or had a travel history to) Hungary (23.5%), Spain (11.3%) and Croatia (7.0%), all three countries endemic to canine dirofilariosis. Interestingly, all *D. immitis*-positive dogs found in the present study originated from Hungary, which is also one of the countries with the highest prevalence for dirofilariosis in Europe [26]. A survey of the literature revealed quite similar study results in other countries. In two studies from Germany, most cases of *D. immitis* were imported from Spain, Greece, Hungary and Italy, while in a retrospective study from Austria, most infected dogs originated from Hungary or Greece [5,27,28]. Likewise, consistent with the data collected in the present study, the incidence and geographical distribution of heartworm disease in Hungarian dogs have risen since 2012 [29]. The countries mentioned above are neighboring countries of Austria or are frequently visited travel destinations, and are also strongly represented among animal welfare organizations that bring shelter and stray dogs to Austria. The increasing number of dogs matching these criteria is comparable to the situation in Germany, where an increasing number of dogs imported from abroad or traveling to other European and non-European countries has also been observed [30]. In addition, an increasing spread of dirofilarial infections can be observed in other countries, such as Greece, Turkey, Bulgaria and Romania [23,31,32].

None of the dogs examined in the present study showed microfilaremia. As the screening for microfilariae in native blood smears shows a sensitivity of 80–89%, this outcome might be due to occult infections, infection with male worms only, or, even more likely, applied prophylactic microfilaricidal therapy [15,33], as was reported from all animal shelters in this study. The sensitivity of the test method also depends on the microfilariae density. The sensitivity of the modified Knott test is even higher than in the native blood smear. In the present study, the use of a native blood smear was found to be sufficient, as PCR was additionally performed [34,35,36]. Of the 111 dogs screened with ELISA antigen-testing, eleven dogs tested positive for *D. immitis*. Only one sample also showed a positive test result in the PCR test. No false-negative ELISA results were found in the PCR screening. These results are consistent with the literature, showing that various in-house test systems based on ELISA antigen-testing have an average sensitivity of up to 84% and a specificity of about 97%, and are suitable for diagnosis even in dogs with a low worm burden [37,38,39]. According to the manufacturer’s data, the SNAP 4Dx Plus test (SNAP 4Dx Plus-Test, IDEXX GmbH, Kornwestheim, Germany), which is frequently used in Austria and was also used in the present study, shows a sensitivity of 99.0% and a specificity of 99.3% [40]. As treatment with macrocyclic lactones can lead to the formation of antigen-antibody complexes in some patients, masking the antigen and resulting in false-negative test results, ELISA antigen-testing was repeated in negative samples, using heated serum [41,42]. Screening for *Dirofilaria* spp. using PCR showed a sensitivity and specificity of 100% in various studies [43,44,45]. Therefore, false-negative results in the PCR screening are very unlikely to occur. However, as can be seen in the present study, the PCR test can be negative in dogs pretreated with milbemycin, resulting in no microfilariae circulating in the blood at the time of sampling, even though infection with macrofilariae could be detected by the ELISA antigen-test. Therefore, as most screening systems can be prone to false-negative (or false-positive) results, thorough screening using a combination of multiple diagnostic tools is recommended [46,47]. The overall surveyed prevalence of *D. immitis* in the population investigated was 9.6%. These results are not to be neglected despite the presence of a certain bias due to the underlying study design recruiting a high proportion of dogs in the sample group from abroad. The observed prevalence is comparable to other—already endemic—countries, such as Italy (8%), France (6.9%) and Hungary (11.3%), and is considerably higher than in the neighboring country Germany, or even—the also endemic country—Croatia (0.4%) [23]. Additionally, although all dogs included in this study were at least six months old (to take the incubation period into account), not all dogs had been located in Austria for an equivalently long period. Therefore, it cannot be excluded that some of the dogs tested (false-) negative during their incubation period despite being infected with *D. immitis*, and the actual prevalence in the study group may have been higher.

Clinical staging in the infected dogs resulted in eight dogs without clinical signs or with mild signs (stage one), three dogs showing clinical signs consistent with stage two and no dogs in stage three. Macrofilariae could be detected in the right ventricle or pulmonary arteries in three dogs by echocardiography. These results are consistent with the literature, noting many altogether asymptomatic cases of canine dirofilariosis and a long, symptom-free incubation period (which are also part of the problem in successful monitoring and prevention of the introduction of *D. immitis* to non-endemic countries) [17,48]. Interestingly, no abnormalities were found in any of the radiographs of the infected dogs, even though, according to literature, approximately 85% of dogs infected with *D. immitis* show radiographically more or less specific pathologies in the area of the heart and lungs [48,49].

In the present study, ten different mosquito species were identified among the 205 individuals trapped during this study period (*Ae. japonicus*, *Ae. vexans*, *An. maculipennis* complex, *An. plumbeus*, *Cq. richardii*, *Cx. pipiens/torrentium*, *Cs. longiareolata*, *Oc. caspius*, *Oc. excrucians* and *Oc. geniculatus*). Of these, seven species are known or are under discussion to be competent vectors for *Dirofilaria* spp. (*Ae. vexans*, *An. maculipennis* complex, *An. plumbeus*, *Coquillettidia* sp., *Cx. pipiens/torrentium*, *Cs. longiareolata*, *Oc. caspius*) and accounted for 98.5% of all mosquitoes trapped [1,15,50,51]. Potential competent mosquito vectors were found at all shelter sites, including three animal shelters housing *D. immitis*-positive dogs, which would theoretically enable transmission from the dogs to a vector during a blood meal. Nevertheless, there was no filarial DNA detected in the mosquitoes screened via PCR in this study. So far, DNA of *D. repens* has been examined in pools of *An. algeriensis* and *An. maculipennis* complex in Austria near the Hungarian border, and no DNA of *D. immitis* has been found to date [11]. Still, the noticeable increase in the prevalence of dirofilariosis in imported shelter dogs is a serious risk factor for endemisation, particularly because of the presence of several mosquito species in Austria potentially able to transmit *Dirofilaria* spp. and the relatively frequent occurrence of these species. In addition, more vector mosquitoes can be expected in Central and Northern Europe in the future due to the advancing climate change. In Austria, the endemisation of *D. repens* already appears to be concluded and could be considered a model for the expected future distribution of *D. immitis,* as the heartworm seems to be spreading mainly to the areas where *D. repens* has already established itself [1,4,8,9].

A limiting factor of the present study is the relatively infrequent set-up of the mosquito traps (once a month for 24 h throughout the main mosquito season of four months), which resulted in a relatively small sample size (*n* = 205). Furthermore, it must be considered that, according to the animal shelters’ managements, all dogs included in this study were regularly treated prophylactically with macrocyclic lactones (milbemycin oxime), which almost excluded the transmission of microfilariae to mosquitoes during a blood meal, since macrocyclic lactones have a microfilaricidal effect. This effect is also seen in the present study, with three dogs showing negative PCR results despite being tested positive for macrofilariae by ELISA antigen-testing.

The results of the present study highlight that the risk of endemisation of canine cardiopulmonary dirofilariosis in Austria (and other non-endemic countries in a similar situation) must be taken very seriously. As unnoticed cases of dirofilarial infections are very common in dogs due to a long prepotency and many asymptomatic courses, the zoonotic potential of *D. immitis* should not be underestimated [52,53]. The risk of human infection while traveling to endemic regions, by infection by one’s own dog, if infected, or infected dogs in the surrounding neighborhood, and the potential severity of human cases should therefore be communicated to the public more strongly. Furthermore, frequent screening of mosquitoes for *Dirofilaria*, as well as other mosquito-borne diseases, is an essential tool for monitoring the progression of this process and should be used more intensively. Regular application of microfilaricides could help prevent transmission from infected dogs to mosquitoes. The authors also recommend a comprehensive integration of current knowledge on prophylactic, diagnostic and therapeutic measures, as published by the American Heartworm Society (AHS), the European Society of Dirofilariosis and Angiostrongylosis (ESDA) and the European Scientific Counsel Companion Animal Parasites (ESCCAP), into the daily practice of veterinarians. The proactive education of dog owners, especially before traveling to endemic areas or adopting dogs from endemic countries, should be enforced. Ultimately, animal shelters and animal welfare organizations should be provided with appropriate education and veterinary guidance regarding the testing and, if necessary, treatment of dogs imported from high-risk areas.

## 4. Materials and Methods

### 4.1. Dogs

A total of 115 shelter dogs from fourteen volunteering animal shelters located in five different Austrian federal states (Burgenland, Lower Austria, Salzburg, Styria and Upper Austria) were examined. Data were collected regarding the age, sex and breed of the dogs, their country of origin and travel history and the presence of clinical signs of dirofilariosis. The clinical examination included examination of general demeanor, skin and hair coat, mucous membranes, capillary refill time, pulse, respiration, auscultation of heart and lungs, body temperature, inspection of the mouth and throat and elicitation of cough. The identification microchip of each dog was scanned if present to ensure distinctive identification. To take the incubation period into account and avoid false-negative test results, only dogs older than six months were included in the study. ELISA antigen-testing could be performed in all 115 dogs and PCR testing in 111 dogs. The survey was approved by the institutional ethics committee (Veterinary University Vienna, Vienna, Austria) and the Austrian Ministry for Education, Science and Research (BMBWF-68.205/0009-V/3b/2019).

### 4.2. Sample Collection

Approximately six milliliters of blood were taken via the cephalic or saphenous vein from all dogs examined, and two milliliters were placed into each of one K3EDTA-, lithium heparin- and Z-Serum Clot Activator-Vacuette (VACUETTE^®^, Greiner Bio-One International GmbH, Kremsmünster, Austria).

### 4.3. Blood Smears

Blood smears were prepared from the K3EDTA whole blood samples using one drop of blood per slide, which was then air-dried, fixed with alcohol and stained with hematoxylin–eosin. Any supernatants of staining solution were removed under running tap water and the smear was air-dried again. The stained blood smears were microscopically (Alphaphol-2 YS2, Nikon Corporation, Tokyo, Japan) screened at a hundredfold magnification and examined for microfilariae.

### 4.4. ELISA Antigen-Testing

ELISA antigen-testing was performed in all 115 dogs. In five dogs, the shelter staff performed the ELISA tests themselves and no further blood tests were obtained. In eight dogs, the ELISA antigen-test was conducted via an external laboratory (LABOKLIN GMBH & CO.KG, Bad Kissingen, Germany). For the remaining 102 dogs, the Z-Serum Clot Activator-Vacuettes were centrifuged in a Hettich-centrifuge (EBA 3S, Andreas Hettich GmbH & Co.KG, Tuttlingen, Germany) for seven minutes at 3000 rpm. The serum was then removed from the sample and stored at −20 °C. Sampling for *D. immitis*-antigen was performed with an ELISA rapid test kit according to the manufacturer’s instructions (SNAP 4Dx Plus Test, IDEXX Laboratories, Westbrook, ME, USA). The dogs that tested negative in the rapid test kit using unheated serum samples were re-tested using heated serum to exclude false-negative results. To break up potential antigen–antibody complexes, the serum was heated for ten minutes at 103 °C, using a heating block (Dry Blockheater 2, IKA^®^-Werke GmbH & CO. KG, Staufen im Breisgau, Germany) and centrifuged for another ten minutes in an Eppendorf centrifuge (Centrifuge 5415 D, Eppendorf AG, Hamburg, Germany).

### 4.5. PCR

PCR testing was performed in samples of 105 dogs. Eight samples were tested via PCR in an external commercial laboratory (LABOKLIN GMBH & CO.KG, Bad Kissingen, Germany). From 97 samples, DNA was extracted using a genomic DNA extraction kit (High Pure PCR Template Preparation Kit, Roche Diagnostik GmbH, Mannheim, Germany). The isolated DNA was then screened twice for the presence of filarioid DNA with conventional PCR, applying two different protocols using Taq polymerase (GoTaq, Promega GmbH, Walldorf, Germany) plus (1) the COI-primers H14FilaCOI-Fw-5′-GCCTATTTTGATTGGTGGTTTTGG-3′ and H14FilaCOI-Rv-5′-AGCAATAATCATAGTAGCAGCACTAA-3′ and (2) the COI-primers COIint-F-5′-TGATTGGTGGTTTTGGTAA-3′ and COIint-R-5′-ATAAGTACGAGTATCAATATC-3′ [17,18].

### 4.6. Mosquitoes

#### 4.6.1. Collection and Classification

Mosquitoes were caught at five different dog shelter sites in four different Austrian federal states (Burgenland, Lower Austria, Salzburg and Styria), using CO_2_-baited mosquito traps set up once a month (June–September 2018) for 24 h (BG-Sentinel, Biogents AG, Regensburg, Germany). The trapping sites selected were protected from wind, rain and sun and located within a maximal distance of five meters from the shelter dogs. The mosquitoes were then pooled by species following the identification keys by BECKER 2010 [54].

#### 4.6.2. PCR

DNA was extracted following the DNeasy^®^ Blood & Tissue Handbook (DNeasy Blood & Tissue Kits, QIAGEN GmbH, Hilden, Germany) and screened for *Dirofilaria* spp. with a touchdown-PCR using Taq polymerase (GoTaq, Promega GmbH, Walldorf, Germany) and the COI-primers COIint-F-5′-TGATTGGTGGTTTTGGTAA-3′ and COIint-R-5′-ATAAGTACGAGTATCAATATC-3′ [55].

## Figures and Tables

**Table 1:** Countries of origin of the examined dogs.

Country of Origin *	Number of Dogs (%)
unknown	21 (18.3)
*Spain*	13 (11.3)
*Romania*	4 (3.5)
*Greece*	2 (1.7)
Austria	27 (23.5)
*Hungary*	27 (23.5)
*Slovakia*	3 (2.6)
*Slovenia*	1 (0.9)
*Croatia*	8 (7.0)
*Serbia*	3 (2.6)
Germany	4 (3.5)
Czech Republic	2 (1.7)

* Countries endemic to *D. immitis* are written in italics.

**Table 2:** Test results of dogs positive in at least one assay.

Sample No. (Location)	Country of Origin	*Dirofilaria immitis Antigen*	Filarioids
		ELISA	PCR
91	Hungary	+	−
103	Hungary	+	−
104	Hungary	+	−
105	Hungary	+	/
106	Hungary	+	/
107	Hungary	+	/
108	Hungary	+	−
112	Hungary	+	−
113	Hungary	+	/
114	Hungary	+	/
115	Hungary	+	+

“+” = positive result; “−” = negative result; “/” = no test performed.

**Table 3:** Mosquitoes caught in July–September 2019 listed by location.

Location	Number of Mosquitoes Caught (Number of Competent Vector Mosquitoes)					
	June	July	August	September	Total	Dogs Positive for *D. immitis* Present at Location
1 (Hallein, Salzburg)	5 (5)	7 (7)	14 (14)	8 (8)	34 (34)	yes
2 (Graz, Styria)	5 (5)	7 (7)	3 (2)	1 (1)	16 (15)	no
3 (Vienna, Vienna)	43 (41)	56 (56)	1 (1)	3 (3)	103 (101)	yes
4 (Murtal, Styria)	2 (2)	0	3 (3)	1 (1)	6 (6)	no
5 (Neusiedl am See, Burgenland)	0	19 (19)	10 (10)	17 (17)	46 (46)	yes
total	55 (53)	89 (89)	31 (30)	30 (30)	205 (202)	

**Table 4:** Species classification of the examined mosquitoes.

Species	Number
*Aedes japonicus*	1
*Aedes vexans*	10
*Anopheles maculipennis* complex	2
*Anopheles plumbeus*	2
*Coquillettidia richardii*	1
*Culex pipiens/torrentium*	185
*Culiseta longiareolata*	1
*Ochlerotatus caspius*	1
*Ochlerotatus excrucians*	1
*Ochlerotatus geniculatus*	1

## Data Availability

Not applicable.
